# Transcatheter Self-Expandable Aortic Valve (Portico) Implantation in a Patient with Previous Mitral Valve Replacement: A Case Report

**Published:** 2019-04

**Authors:** Ahmet Korkmaz, Havva Tuğba Gürsoy, Mehmet İleri, Özgül Uçar Elalmış, Ümit Güray

**Affiliations:** *Department of Cardiology, Ankara Numune Training and Research Hospital, Ankara, Turkey.*

**Keywords:** *Aortic valve stenosis*, *Transcatheter aortic valve replacement*, *Mitral valve*

## Abstract

Transcatheter aortic valve implantation (TAVI) has shown favorable outcomes in patients with severe symptomatic aortic valve stenosis who are at high surgical risk or who are unsuitable candidates for open-heart surgery. However, concerns exist over treating patients who have undergone previous mitral valve surgery due to the potential interference between the mitral prosthetic valve or ring and the TAVI device. In this case report, we present a case in which a patient with symptomatic severe aortic stenosis and previous mechanical mitral valve replacement was successfully treated with TAVI using a Portico valve, which is under-researched**.**

## Introduction

Aortic stenosis is one of the most common heart valve diseases in the developed world, and the only definitive treatment for severe symptomatic aortic stenosis is aortic valve replacement. Open-heart surgery is still the gold standard in the treatment of low-risk patients with aortic stenosis; and in the last 15 years, transcatheter aortic valve implantation (TAVI) has proven to be superior or at least non-inferior in moderate or high operative risk patients.^1^^-^^3^ In general, reoperations after previous cardiac surgery are associated with increased mortality and a high risk of adverse events. Thus, a history of previous cardiac surgery in patients with symptomatic severe aortic stenosis is considered to be an indication for TAVI. However, we should note that there are still concerns relating to the treatment of patients who have undergone previous mitral valve surgery due to the proximity and the potential mechanical interference between the TAVI device and the previously implanted mitral prosthesis. There are only a limited number of reports on the feasibility of TAVI using a Portico (St. Jude Medical, St. Paul, MN, United States) instead of a CoreValve (Medtronic, Minneapolis, MI, United States) or a SAPIEN (Edwards Lifesciences – Irvine, CA, United States) in patients with a prosthetic mitral valve. In this case report, we present a patient with symptomatic severe aortic stenosis who had previously undergone a mechanical mitral valve replacement and who was successfully treated with TAVI using a Portico.

## Case Report

**Figure 1:**
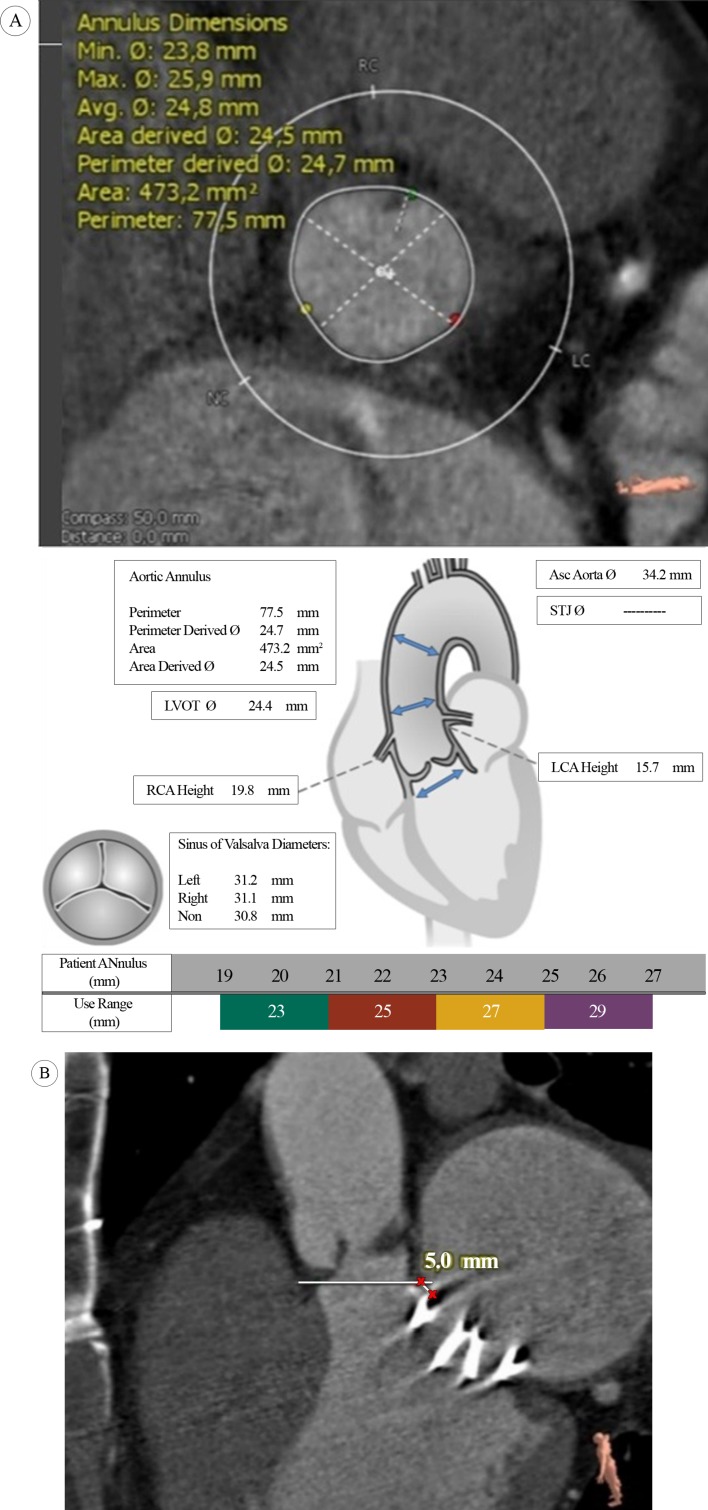
A) Computed tomography images of a calcified aortic valve and aortic annulus with valve sizes; and B) minimum distance between the prosthetic mitral valve and the aortic valve annulus

LVOT, Left ventricular outflow tract; RCA, Right coronary artery; LCA, Left coronary artery

 A 53-year-old male patient presented with complaints of shortness of breath and chest pain due to severe aortic valve stenosis diagnosed in a local hospital. The patient had a medical history of mitral valve replacement with a mechanical prosthetic valve in 2011. The electrocardiogram (ECG) on admission showed sinus rhythm and findings suggestive of left ventricular hypertrophy, while transthoracic echocardiography showed a heavily calcified aortic valve with an aortic valve area of 0.81 cm^2^ and peak and mean pressure gradients of 87 and 55 mmHg, respectively. The bileaflet mechanical mitral valve was intact. The left ventricle showed normal systolic function and dimensions with mild concentric hypertrophy. Mild pulmonary hypertension was identified with a right ventricular systolic pressure of 44 mmHg and mild tricuspid regurgitation. Computed tomography (CT) images demonstrated a calcified aortic valve with an annulus diameter that ranged from 23.8 to 25.9 mm, an annulus perimeter of 77.5 mm, and an annulus area of 473.2 mm^2^ (Figure 1A).The minimum distance between the prosthetic mitral valve and the aortic valve annulus was 4.5 to 5 mm (Figure 1B). A CT angiography identified iliac arteries with a diameter greater than 7 mm and common femoral arteries with a diameter greater than 6.5 mm on both sides. No significant stenosis or calcification was noted in the iliac or common femoral artery. Coronary angiography revealed no significant stenosis. The logistic EuroSCORE II was calculated to be 12.8%, and the Society of Thoracic Surgeons (STS) score was 2.6. A multidisciplinary heart team evaluated the case and confirmed TAVI in the patient.

**Figure 2:**
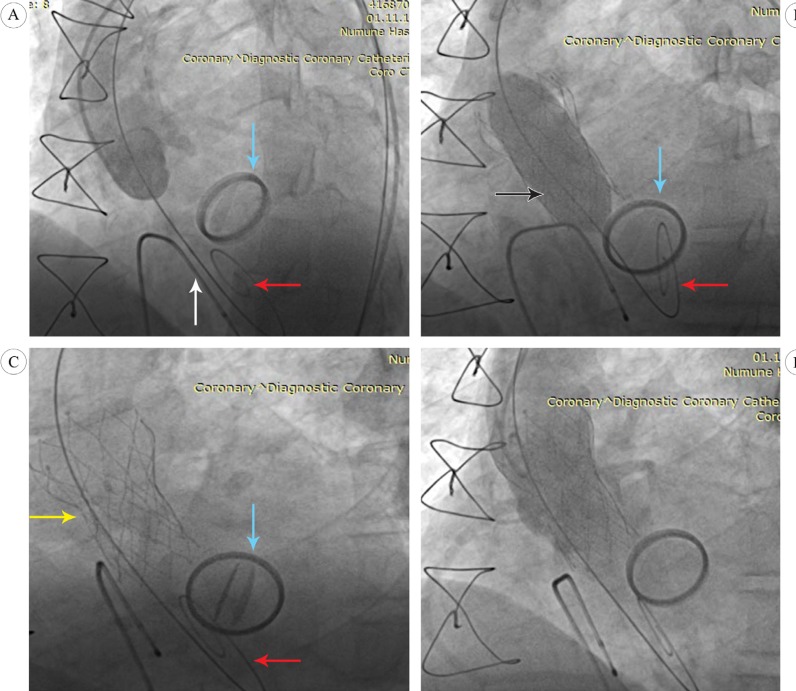
Fluoroscopic images of transcatheter aortic valve implantation using a Portico

A) Pre-implantation aortography and angiographic assessment of the aortic annulus to the prosthetic mitral valve; B) post-dilation using a 25-mm balloon, performed under rapid pacing; and C) & D) fluoroscopic and aortic root angiography, showing a mild paravalvular leak and an adequate positioning of the implanted Portico, and the prosthetic mitral valve, showing intact valve function

Black arrows, 25 mm balloon; Blue arrows, Prosthetic mitral valve; Yellow arrows, Portico valve; Red arrows, Safari wire; White arrows, Temporary pacemaker lead

The TAVI procedure was performed in the catheter laboratory room under local anesthesia and sedoanalgesia. The vascular access for the Portico delivery catheter was created in the right common femoral artery using standard percutaneous access techniques. The “Perclose ProGlide” technique was applied before the insertion of the sheath to prepare pre-tied knots for the closure of the puncture site after the procedure. A temporary pacemaker was placed in the right ventricle via the left femoral vein, and a 6-F pigtail catheter was inserted into the ascending aorta via the left common femoral artery for angiographic and anatomic guidance (Figure 2A). Then, a 0.038-inch wire was passed through the aortic valve using a 6-F AL1 catheter and exchanged with the pigtail catheter and subsequently, a pre-shaped Safari wire. A delivery sheath loaded with a 27-mm Portico valve was inserted through the aortic valve without predilation, and the position of the Portico was adjusted within the aortic valve, avoiding contact with the prosthetic mitral valve, and was slowly deployed under angiographic and fluoroscopic guidance. After the placement of the valve, aortic root angiography revealed a mild-to-moderate grade of a paravalvular leak. Thus, a postdilation using a 25-mm balloon was performed under rapid pacing (Figure 2B). A subsequent angiography showed a decreased paravalvular leak (mild AR), and an adequate positioning of the implanted Portico and the prosthetic mitral valve showed intact valve function (Figures 2C & 2D). The peak and mean pressure gradients over the aortic valve, as measured by echocardiography, were decreased from 87 to 18 mmHg and 55 to 8 mmHg, respectively. The vascular access was closed without complications, and the patient was monitored in an intensive care unit for 2 days. No conduction abnormalities were observed on ECG. On the fifth postprocedural day, the patient was discharged without any significant complications. His symptoms subsequently improved from New York Heart Association (NYHA) class II-III to class I. 

## Discussion

Patients with an estimated mortality risk of greater than 10% by the logistic EuroSCORE or greater than 8% according to the Society of Thoracic Surgeons score system are considered candidates for TAVI procedures.^4^ Combined respiratory failure; pulmonary hypertension; previous cardiac surgery; right ventricular failure; hostile thorax caused by occurrences such as radiation, burns, previous thoracic pleurodesis, and multiple thoracotomies; severe connective tissue disease; liver cirrhosis; cachexia; and porcelain aorta are further indicators of TAVI.^4^^, ^^5^ A history of cardiac surgery is associated with increased mortality (5%–26%) due to postoperative adhesion and the patient’s impaired general condition.^6^^, ^^7^

Technical concerns related to TAVI in patients with prosthetic mitral valves are centered on the potential interactions between the aortic and mitral prostheses at the anatomic aorto-mitral continuity. A significant reduction or even the absence of mitroaortic space to accommodate the balloon-expanded valve and the presence of a mechanical structure instead of fibrous tissue can limit the expansion of the percutaneous valve.^8^^, ^^9^ Hence, a pre-existing prosthetic valve or ring was considered an exclusion criterion in clinical trials such as the PARTNER II trial (Edwards SAPIEN valve) and the Medtronic CoreValve U.S. Pivotal Trial.^10^^, ^^11^

Despite initial concerns, the feasibility of TAVI in the presence of a mechanical prosthesis has been demonstrated. However, experience regarding the feasibility of TAVI to date is limited. In addition to other possible complications after TAVI such as prosthesis embolization, coronary artery obstruction, cardiac tamponade, embolic events, renal failure, and heart block, there is a chance of mechanical valve dysfunction, either intra- or postprocedurally.^12^

Several reports have demonstrated that TAVI can be successfully performed in patients with mechanical or biological mitral valves or with annuloplasty mitral rings.^12^^-^^14^ However, there have been only a limited number of reports on TAVI using the Portico, a self-expandable stent with a longer stent frame than in balloon-expandable valves, in patients with a mechanical mitral valve.

We decided to implant a Portico TAVI system for the following reasons. Firstly, the Portico system is designed to function at the level of the annulus, conferring time to evaluate mitral valve function before full TAVI deployment. Secondly, the large cell-frame design of the Portico could theoretically minimize the risk of interference within the mitral valve leaflets. Thirdly, the possibility of the resheathing and redeploying of the valve is of great significance in previously implanted prosthetic mitral valves.

In the present case, we avoided any displacement or deformation of the Portico and the distortion of the pre-existing mitral prosthesis by meticulously positioning the TAVI device under fluoroscopy guidance, and the postprocedural echocardiography revealed intact mitral valve function. A preprocedural assessment using echocardiography and CT images is important if potential interference between the aortic annulus and the mitral prosthesis is to be avoided. The intraprocedural imaging guidance by transesophageal echocardiography or fluoroscopy is critical when adjusting the extent of the prosthetic valve strut protrusion into the left ventricular outflow tract. Our case demonstrates that TAVI using a Portico can be carried out safely in patients with previously implanted prosthetic mitral valves.

## Conclusion

TAVI in patients with a pre-existing mitral prosthesis is feasible, but prospective long-term follow-up data are needed.

## Notes:

***This paper should be cited as: ***Korkmaz A, Gürsoy HT, İleri M, Uçar Elalmış Ö, Güray Ü. Transcatheter Self-Expandable Aortic Valve (Portico) Implantation in a Patient with Previous Mitral Valve Replacement: A Case Report. J Teh Univ Heart Ctr 2019;14(2):85-89.
